# Mandibular Osteitis Fibrosa Cystica as First Sign of Vitamin D Deficiency

**DOI:** 10.1155/2018/6814803

**Published:** 2018-04-15

**Authors:** Nour Mellouli, Raouaa Belkacem Chebil, Marwa Darej, Yosra Hasni, Lamia Oualha, Nabiha Douki

**Affiliations:** ^1^Oral Medicine and Oral Surgery, Dental Department, University Hospital Sahloul, Sousse, Tunisia; ^2^Oral Health and Oro-Facial Rehabilitation Laboratory Research (LR12ES11), Faculty of Dental Medicine, University of Monastir, Avenue Avicenne, 5019 Monastir, Tunisia; ^3^Endocrinology Department, University Hospital Farhat Hached, Sousse, Tunisia; ^4^Endodontics, Dental Department, University Hospital Sahloul, Sousse, Tunisia

## Abstract

**Introduction:**

Brown tumors of hyperparathyroidism are locally destructive bone lesions. They are the late clinical consequence of the disease. They can occur in primary, secondary, and rarely tertiary forms. They affect usually long bones and less frequently those of the maxilla.

**Case Report:**

Our 45-year-old female patient presented with a mandibular tumor next to the first right lower molar. At first, we have chosen tooth extraction and tumor excision. When the histological report showed the giant cell tumor we suspected a metabolic bone disorder. Biochemical tests screened hyperparathyroidism and severe vitamin D deficiency, and parathyroid scintiscan revealed parathyroid adenoma.

**Discussion:**

The association of hyperparathyroidism and vitamin D deficiency leads to diagnostic uncertainty. First, secondary hyperparathyroidism can be due vitamin D deficiency. Second, data available show that vitamin D deficiency is more prevalent in patients with primary hyperparathyroidism than in general population. Hyperparathyroidism management is based on correct and precise diagnosis. Furthermore, the resolution of brown tumors depends on the cure of hyperparathyroidism. In fact, bone lesions should regress after biological tests' normalization.

**Conclusion:**

Clinicians should be aware of such rare and complicated presentation. They must consider the diagnosis of the brown tumor to avoid extensive surgical excision and teeth extractions.

## 1. Introduction

Hyperparathyroidism (HPT) is a prevailing endocrine disease. Determined by the cause of PTH production, HPT can be characterized into primary, secondary, and tertiary form(s). Primary hyperparathyroidism (PHPT) is a disorder of calcium, phosphate, and thus bone metabolism. The main cause of PHPT is adenoma in about 80% of cases followed by glandular hyperplasia (15%) and more rarely due to the presence of parathyroid carcinoma. In primary HPT, hypercalcemia and hypophosphatemia are omnipresent in laboratory tests [1]. Secondary hyperparathyroidism (SHPT) is caused by defective phosphate excretion and failure to activate vitamin D. Elevated phosphate level, decreased calcium level, and reduced serum vitamin D lead to continuous stimulation of the parathyroid glands that increases PTH release [1].

Tertiary hyperparathyroidism is a state of excessive secretion of PTH after a long period of SHPT. It leads to autonomous parathyroid glands that induce hypercalcemia [1].

HPT leads to bone involvement that includes generalized osteoporosis, multiple focal skull areas of demineralization, and brown tumors.

Brown tumors affect usually clavicles, ribs, and pelvis. Head and neck involvement is rare, and the mandible is affected more often than the maxilla.

The aim of this paper was to describe a case of the brown tumor in the mandible which was the first sign of hyperparathyroidism.

## 2. Case Report

**Figure 1:**
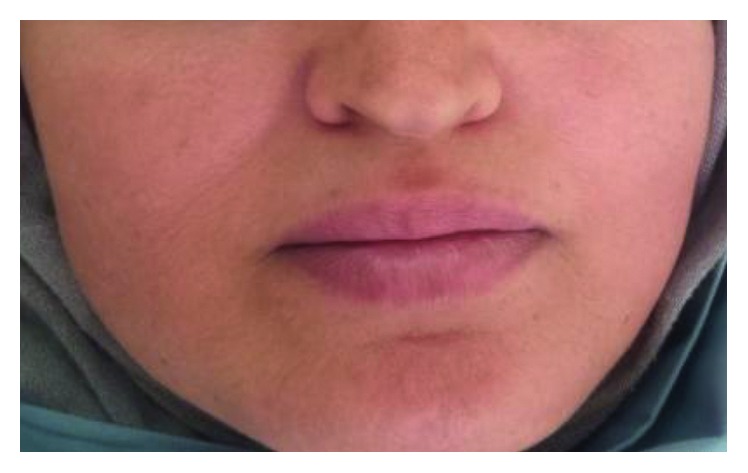
Clinical picture: slight asymmetry with swelling on the right side of the face.

A female patient aged 45 years with no known comorbidities came to our department at the university hospital Sahloul Sousse with the chief complaint of a right-sided swelling in the mandible. This swelling has caused slight asymmetry of the face since 6 months, which gradually enlarged up to the present size (Figure 1).

The patient gave a history of generalized weakness, lethargy, and weight loss noticed since past few months. Her family history and past medical history were nonsignificant.

**Figure 2:**
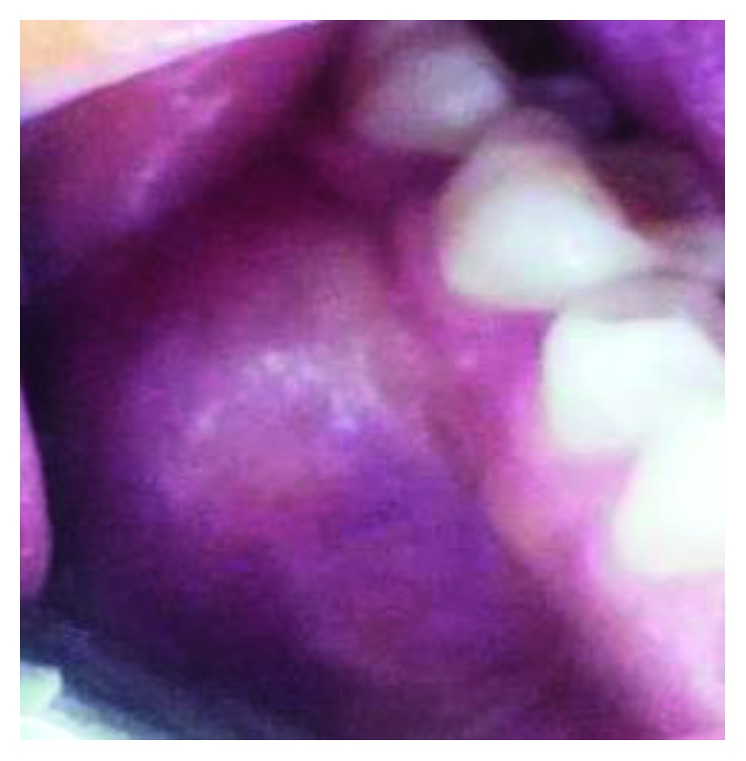
Clinical picture: a bulbous mass of 4 × 4 cm in the vestibule on the right side, extending from the distal aspect of 44 to the 47.

Intraoral examination revealed a 4.0 × 4.0 cm bulbous mass hard to palpation arising from the mandible and extending from the distal aspect of the lower right first premolar (44) to the second right molar (47) (Figure 2). She had no associated bleeding or superficial ulceration of the mass. The patient denied pain but mentioned difficulty in eating. Positive response to sensitivity testing was found in the lower right premolars and molars. Neither mobility nor teeth dislocation were noticed.

**Figure 3:**
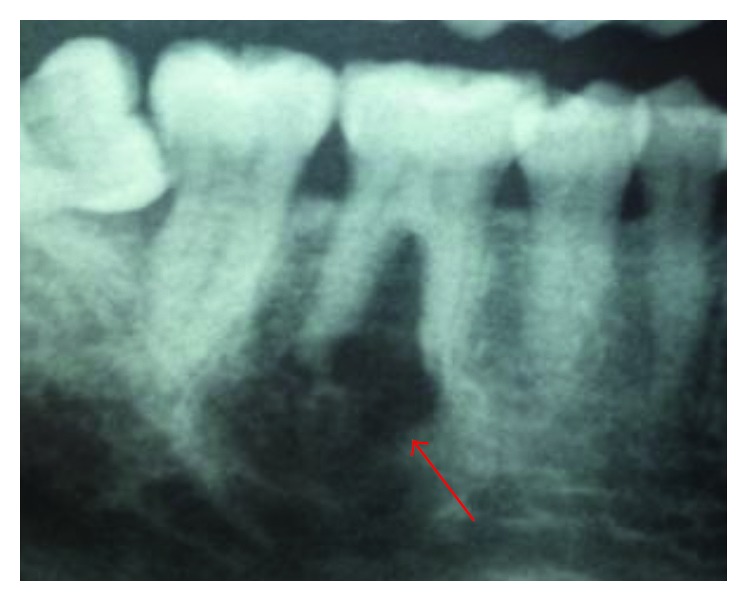
Panoramic radiography showing unilocular radiolucency close to the 46.

Radiographic examination with orthopantamogram showed unilocular radiolucency close to the mandibular first right molar's roots, without involving the mandibular canal. Roots erosion of the relative tooth was noted (Figure 3).

**Figure 4:**
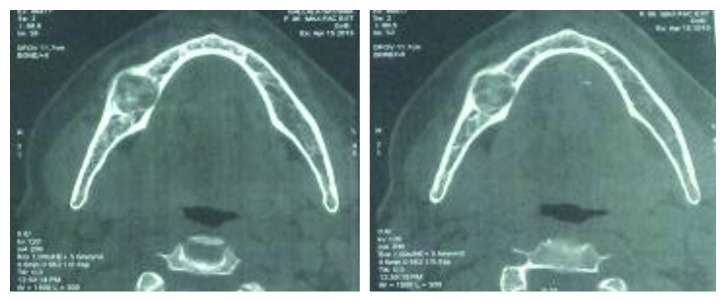
Computed tomography scan: axial views showing multiloculated ground-glass ossification of the lesion.

A computed tomography (CT) scan showed multiloculated ground-glass ossification of the lesion (Figure 4).

**Figure 5:**
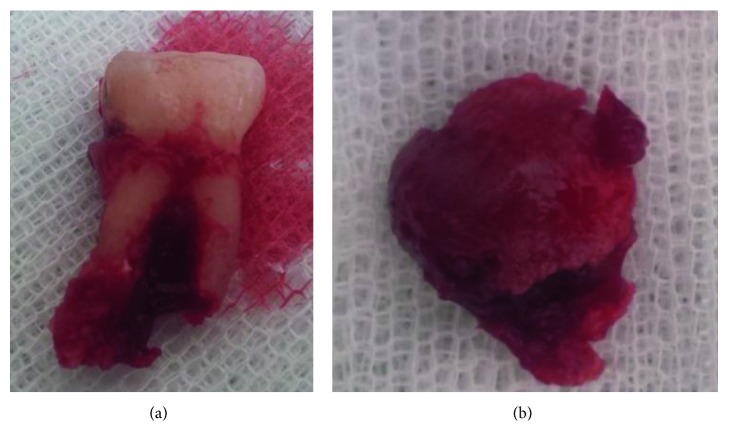
Biopsy specimen.

After the first molar extraction (46) and the surgical excision of the lesion, the histological report was akin to the giant cell tumor (Figure 5).

So, serum parathormone level was advised to rule out metabolic bone disease. Laboratory investigations were done: serum PTH level was slightly high, and serum calcium and phosphorus levels were normal (Table 1).

The lesion was identified as a brown tumor of hyperparathyroidism, and the patient was referred to the endocrinology department.

Kidney function blood tests were done. Serum creatinine and blood urea nitrogen were normal. Chronic renal failure could not be considered the cause of hyperparathyroidism. However, we noted severe vitamin D deficiency (Table 1). The initial impression was of secondary hyperparathyroidism due to this vitamin D deficiency.

It was decided that vitamin D supplementation was the best therapeutic option. The patient was started on vitamin D 100,000 IU per fortnight for 6 weeks which is the equivalent of half an ampoule of 200,000 IU/1 ml. Thereafter, the patient was kept on 200,000 IU every 6 months. Her PTH levels decreased after 6 months on vitamin D therapy, and her calcium and phosphorus levels remained in the standards.

**Figure 6:**
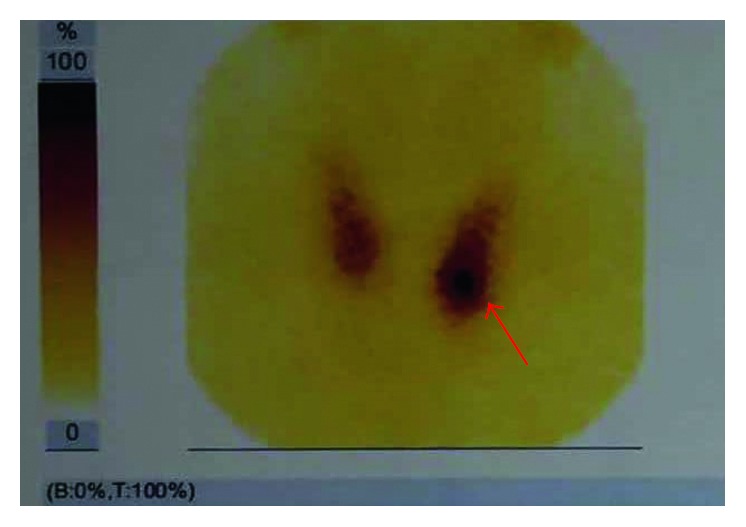
Parathyroid technetium scintiscan (99mTc Sestamibi; Technetium-99 MIBI; methoxy-isobutyl-isonitrile) showed increased uptake of the radiocontrast agent observed in the left lower parathyroid.

The patient underwent ultrasound scan of the neck, which showed a left lower lobe parathyroid solid nodule. The lesion had irregular hypoechoic component and was suggestive of parathyroid adenoma. Parathyroid technetium scintiscan (99mTc Sestamibi; Technetium-99 MIBI; methoxy-isobutyl-isonitrile) was requisite and revealed left lower parathyroid adenoma (Figure 6). Skeleton exploration was done by technetium 99 scintigraphy and did not reveal any abnormally high uptake.

Accordingly, we retained the diagnosis of primary hyperparathyroidism masked by vitamin D deficiency and caused by parathyroid adenoma.

**Figure 7:**
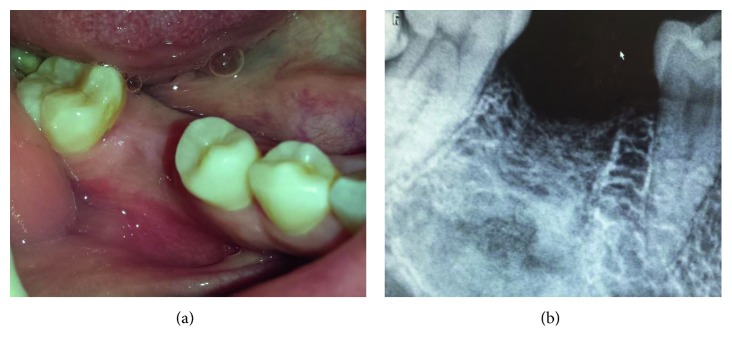
Clinical and radiological control at the 6 month with good mucosal and bone healing.

There was a normalization of calcium, PTH, and vitamin D serum levels. The bone mineral density test was not lower than normal which excludes osteopenia and osteoporosis. So, surgical treatment of the adenoma was not indicated. The patient was compliant and presented a favorable evolution (Figure 7).

## 3. Discussion

Before the 1970s, the presentation of HPT was characterized by recurrent nephrolithiasis, brown tumors, neuromuscular dysfunction, symptomatic hypercalcemia, peptic ulcer disease, psychosis, and pancreatitis [2]. After then, with the introduction of the automated biochemistry analyzer, HPT could be diagnosed in the early and asymptomatic period of this disease [3]. Patients with profound symptoms became rare besides those apparently asymptomatic emerged. For these patients, elevated serum PTH level was discovered incidentally.

The brown tumor also known as osteitis fibrosa cystica or Von Recklinghausen's disease of bone is a reactive nonneoplastic giant cell lesion associated with hyperparathyroidism. Products from microbleeding like hemosiderin assign the brown color to the lesion [4, 5]. The lesion is more prevalent in patients older than 50 years and three times more frequent in women [6]. The female preponderance is speculated to be due to women's lower body mass that would lead to an earlier clinical manifestation of the disease [7].

The preferential location of brown tumors is ribs, clavicle, and pelvic girdle. When they arise in the maxillofacial region, the mandible is so far the most frequent localization [8]. However, both mandible and maxilla affected individually or simultaneously were reported [7].

The physical examination usually reveals painful and hard bone swelling which may produce disfiguring deformities. However, our patient denied any ache. Asymptomatic brown tumors are also described by several authors. Therefore, their discovery is fortuitous following a radiological examination.

On radiographic and computed tomography exams, well-defined radiolucent or hypodense image are described, usually not demonstrating cortical disruptions and periosteal reactions or inflammatory signs [6]. This typical cyst-like radiographic appearance can be replaced by a multiloculated lesion with a “ground-glass opacification” which was found in our case [4].

Clinical and radiological presentation of the brown tumor can mimic other diseases, the most likely diagnoses include odontogenic and nonodontogenic cysts and tumors (radicular cyst, lateral periodontal cyst, ameloblastoma, keratocyst, eosinophilic granuloma, giant cell lesions, myxoma, and fibroosseous lesions), infectious diseases (bone abscess and localized osteomyelitis), and metastasis from a known or an unknown primary site (lung, breast, kidney, and prostate) [9, 10].

The usefulness of the tumor biopsy is controversial. When biological disorder guides to brown tumor diagnosis, it is useless. Histologically, the presence of diffuse giant cells is characteristic, but there is no pathognomonic sign [4].

Brown tumors occur in 4.5% of patients with PHPT and between 1.5% and 1.7% in cases of SHPT [5, 8] with an overall prevalence of 0.1% [4, 6]. The treatment of brown tumors is the cure of the underlying hyperparathyroidism. When hypersecretion of PTH is corrected, spontaneous regression of the lesion is expected. In our case, precipitated surgery led to loosing tooth and wide bone defect. Then, a proper diagnosis could have avoided inadequate excision and teeth extractions. Surgical therapy is required in a second step if the lesion persists or if bone healing is compromised, but only after HPT is controlled [4].

To correct hyperparathyroidism, it is worthwhile to know its form. Secondary HPT results when hypersecretion of PTH is a response to decreased calcium. It is generally associated with serum hypocalcemia and hyperphosphatemia. This condition is found in patients with chronic kidney disease or vitamin D deficiency [11].

The main cause of primary HPT is parathyroid adenoma. Glandular hyperplasia and parathyroid carcinomas are more rare etiologies. Primary HPT is characterized by elevated or inappropriately normal PTH levels and the persistent elevation of total serum calcium levels [12]. Normocalcemic primary HPT is a variant newly acknowledged. That clinical entity is poorly known and develops with high PTH levels and normal serum calcium levels [12].

The American Association of Endocrine Surgeons recommends the biochemical evaluation of serum total calcium, PTH, creatinine, and vitamin D levels if primary HPT is suspected (strong recommendation and moderate quality evidence) [12].

Low levels of vitamin D are found more often in primary HPT than in the general population. This observation is dyed in the wool [13] and is based upon measurement of the serum 25-hydroxyvitamin D level. Vitamin D deficiency's definition is controversial. Many experts define two groups: “insufficiency” in which the level of serum 25-hydroxyvitamin D is between 20 and 30 ng/mL and the other “deficiency” in which the level is <20 ng/mL [14]. The pathophysiological mechanism explaining the association between vitamin D deficiency and primary HPT is not clear. But chronic vitamin D deficiency seems to incite events leading to parathyroid gland hyperplasia and subsequent adenomatous changes [13].

In our case report, biological findings could go along not only with the secondary HPT but also with the normocalcemic variant of primary HPT. To fix on the diagnosis, neck scintiscan revealed parathyroid adenoma. So, diagnosis of primary HPT was retained.

The supplementation for vitamin D deficiency is required, and maintaining vitamin D to levels beyond 30 ng/ml is recommended. The endocrine society supported vitamin D2 or D3 supplementation of 50,000 IU once a week for 8 weeks or its daily equivalent followed by 1,500 to 2,000 IU daily use maintenance. In our case, the protocol has been modified since only pharmaceutical form of 200,000 IU/1 ml is available [15].

Parathyroidectomy should be considered for most patients with primary HPT and is more worthwhile than observation or pharmacologic therapy. It is the definitive treatment option for all patients with symptomatic primary HPT or associated with osteoporosis. Surgical treatment is also indicated when the serum calcium level is higher than 1 mg/dL (0.25 mmol/l) above normal, even if there are no symptoms. Patients, younger than 50 years, and those unable or unwilling to comply with observation protocols, are rather candidates for surgery [12, 13, 16].

## 4. Conclusion

The incidence of primary hyperparathyroidism is increasing. Surprisingly, it tripled between 1995 and 2010, and clinicians from all specialties will likely encounter patients with this disorder. There is some evidence to suggest that vitamin D deficiency may increase the likelihood of a more symptomatic presentation of PHPT. The exact influence of vitamin D status upon the modern presentation of PHPT is yet to be fully defined.

The treatment of brown tumors is the cure of the underlying hyperparathyroidism. When hypersecretion of PTH is corrected, spontaneous regression of the lesion is expected. A typical radiological presentation of the giant cell tumor should be completed by measurement of calcium, phosphorus, and PTH serum levels. Then, a proper diagnosis avoids inadequate surgical excision and teeth extractions.

## Figures and Tables

**Table 1:** Blood tests of the patient before and after vitamin D supplementation.

Serum level	Normal range	Initial values	After vitamin D therapy (6 months)
PTH (pg/ml)	15–65	81.5	56.3
Calcium (mmol/l)	2.15–2.5	2.3	2.4
Phosphorus (mmol/l)	0.87–1.45	1.3	1.1
25-hydroxyvitamin D (ng/ml)	>(20–30)	9.5	17.9
